# Comparing three UV wavelengths for pre‐exposing Gafchromic EBT2 and EBT3 films

**DOI:** 10.1120/jacmp.v16i6.5663

**Published:** 2015-11-08

**Authors:** Toshizo Katsuda, Rumi Gotanda, Tatsuhiro Gotanda, Takuya Akagawa, Nobuyoshi Tanki, Tadao Kuwano, Kouichi Yabunaka

**Affiliations:** ^1^ Faculty of Human Relation Tokai Gakuin University Gifu Japan; ^2^ Department of Radiological Sciences Ibaraki Prefectural University of Health Sciences Ibaraki Japan; ^3^ Faculty of Health Sciences Junshin Gakuen University Fukuoka Japan; ^4^ Department of Radiological Technology Tokushima Red Cross Hospital Tokushima Japan; ^5^ Center for Life Science Technologies RIKEN Hyogo Japan; ^6^ Graduate School of Health Sciences Okayama University Okayama Japan; ^7^ Graduate School of Medicine the University of Tokyo Tokyo Japan

**Keywords:** ultraviolet rays, nonuniformity, Gafchromic film, radiation measurement, computed tomography

## Abstract

Gafchromic films are used for X‐ray dose measurements during diagnostic examinations and have begun to be used for three‐dimensional X‐ray dose measurements using the high‐resolution characteristics of Gafchromic films for computed tomography. However, the problem of unevenness in Gafchromic film active layers needs to be resolved. Double exposures using X‐rays are performed during therapeutic radiology, although this is difficult for a diagnostic examination because of a heel effect. Thus, it has been suggested that ultraviolet (UV) radiation be used as a substitute for X‐rays. However, the appropriate UV wavelength has not been determined. Thus, we conducted this study to decide an appropriate UV wavelength. UV peak wavelengths of 245 nm (UV‐A), 310 nm (UV‐B), and 365 nm (UV‐C) were used to irradiate EBT2 and EBT3 films. Each UV wavelength was irradiated for 5, 15, 30, and 60 min, and irradiation was then repeated every 60 min up to 360 min. Gafchromic films were scanned after every irradiation using a flatbed scanner. Images were split into RGB images, and red images were analyzed using ImageJ, version 1.44, image analysis software. A region of interest (ROI) one‐half inch in diameter was placed in the center of subtracted Gafchromic film images, and UV irradiation times were plotted against mean pixel values. There were reactions in the front and back of Gafchromic EBT3 and the back of Gafchromic EBT2 with UV‐A and UV‐B. However, UV‐C resulted in some reactions in both sides of Gafchromic EBT2 and EBT3. The UV‐A and UV‐B wavelengths should be used.

PACS number(s): 87.53 Bn

## I. INTRODUCTION

Gafchromic films are used for X‐ray dose measurements during diagnostic radiology, including computed tomography (CT), interventional radiology (IVR), and quality control (QC) and quality assurance (QA). For CT dose measurements, three‐dimensional dosimetry methods have been developed, such as a sheet‐roll phantom^(1)^ or a half cylindrical phantom with Gafchromic films.^(2)^ Gafchromic film uniformity is an important consideration for these high‐resolution measurements. Gafchromic film nonuniformity errors that arise because of the unevenness of active layer thicknesses can affect the measured doses. In the diagnostic range for X‐rays, exposure can be slightly changed based on Gafchromic film density. Thus, signal data for X‐ray exposures are affected by these nonuniformity errors.

A double‐exposure technique is used to reduce these nonuniformity errors.^(3)^ However, it is difficult to provide homogenous X‐ray exposure over a wide area, such as a 14×17 inch format. It is known that Gafchromic films react with certain ultraviolet (UV) rays.^(4)^ Gafchromic films have been used to measure the amount of UV rays using this reaction.^(5)^ In general, when using Gafchromic films, UV irradiation was originally considered to be taboo because it introduces density noise for X‐ray measurements.^(6)^ Nonuniformity errors are expressed using UV irradiation as a substitute for X‐rays in a double‐exposure technique. UV light can be uniformly irradiated over a wide area, and nonuniformity errors can be reduced by a subtraction method. In a preliminary study, we performed UV (360 nm) exposure ($0.018\;\text{mW}/{\text{cm}}^{2}$) as a double‐exposure technique for Gafchromic EBT and obtained good results.^(7)^

Because of the unevenness in the thickness of the active layer that could affect true data to appear as noise, UV rays were irradiated uniformly as a double‐exposure technique with the aim of removing nonuniformity errors. An effect was confirmed for homogeneous improvements in UV irradiation when using Gafchromic EBT,^(7)^ although the effects were unknown for Gafchromic EBT2 and EBT3. In these films, a yellow dye is included in an active layer to reduce nonuniformity errors. A study has shown that the uniformity of Gafchromic EBT2 with a single red channel in a double‐exposure technique (pre‐irradiation technique) is equal to that of a triple‐channel method.^(8)^ Thus, UV could be used for pre‐exposure to improve uniformity. It is still necessary to confirm the reaction of UV rays so that we can determine if this yellow dye and UV exposure enhanced this reduction in nonuniformity errors. However, there is a problem with UV irradiation of Gafchromic films. In particular, the sensitivity to the UV rays of the Gafchromic film active layer needs to be clarified. Because there are different wavelengths of UV rays, ranging from 200 to 400 nm, the most suitable wavelength or an adaptation wavelength needs to be determined. Therefore, the aim of this study was to find suitable UV (A, B, or C) rays at which Gafchromic EBT2 and EBT3 active layer are more reactive.

In general, UV rays are divided into UV‐A, UV‐B, and UV‐C, depending on the UV wavelength.^(9)^ UV‐A wavelengths are from 315 to 400 nm, UV‐B wavelengths are from 280 to 315 nm, and UV‐C wavelengths are from 100 to 280 nm.^(9)^ In this study, we used UV‐A of 365 nm, UV‐B of 310 nm, and UV‐C of 245 nm, and the sensitivities of Gafchromic films were compared.

## II. MATERIALS AND METHODS

### A. Gafchromic films and UV lamps

Peak wavelengths of 365 nm (UV‐A), 310 nm (UV‐B), and 245 nm (UV‐C) of UV light were used to expose two different Gafchromic films. A UV lamp that generated a UV peak wavelength of 365 nm was a black light, NEC FL10SBL (10 W) (NEC Lighting Ltd., Tokyo, Japan). A UV lamp that generated a UV peak wavelength of 310 nm was a UV‐B chemical lamp, FL‐10E (10 W) (Kyokko Denki Co. Ltd., Tokyo, Japan). A sterilization lamp that generated a UV peak wavelength of 254 nm was a Toshiba GL10SBL (10 W) (Toshiba Lighting & Technology Corp., Kanagawa, Japan). Two different Gafchromic films (both front and back sides) were used in this study, Gafchromic EBT2 (Lot # 02171403) and Gafchromic EBT3 (Lot # 04011401) (Ashland Inc., Covington, KY). EBT2 and EBT3 are transverse‐type films; however, reflection‐mode scanning was performed because the nonuniformity errors were small.^(10)^ The front side of a Gafchromic EBT2 film is UV protected, but the back side is not.^(11)^ Gafchromic EBT3 is not a UV protected film.^(12)^

### B. UV exposure

**Figure 1:**
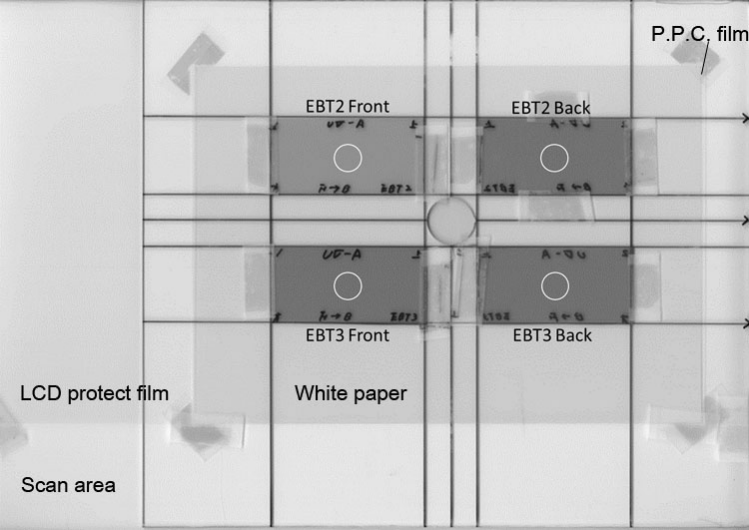
Scanned image of Gafchromic EBT2 and EBT3 film placed on 3 mm acrylic plate. White circles indicate ROIs for measurement of histogram analysis of pixel values.

Our experiments were conducted at night so that there was no interference from solar UV rays. The fluorescent lamps in the room were exchanged with UV ray cutting lamps. The cut work with a Gafchromic film was performed under an incandescent lamp. Using a 245 nm germicidal light, a 310 nm chemical lamp, and a 365 nm black light, because UV‐rays are harmful to the human body,^13^, ^14^ the area surrounding an exposure was protected from UV rays with acrylic plates (Comoglas CG UV40 P, 0.3 cm thickness, Lot # 140406C B) (Kuraray Co. Ltd., Tokyo, Japan). A Gafchromic film was cut to 9 cm (long axis) by 4.5 cm (short axis). Both sides of Gafchromic EBT2 and EBT3 were placed on an acrylic board 3 mm thick (Fig. 1). Because each film was irradiated with each type of UV light, three sets were prepared.

**Figure 2:**
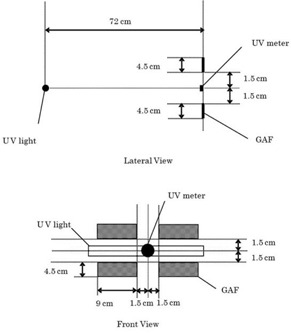
Arrangement for ultraviolet (UV) exposure. Lateral view (top) and frontal view (bottom).

Frontal and lateral view arrangements for UV exposure to Gafchromic films are shown in Fig. 2. The distance between a UV source surface and an optical detector surface was 72 cm. A UV meter probe was placed in the center of an exposure area and UV light strength was measured. UV ray strength was measured using a UVR‐300 with a UD‐360 probe (365 nm) and a UD‐250 (245 nm) probe (Topcon Technohouse Corp., Tokyo, Japan). UV rays of 310 nm were measured by a UV Light Meter UV‐340A (Mother Tool Corp., Nagano, Japan).

### C. Image Scanning

Each UV ray source was used for exposure for 5, 15, 30, and 60 min, and exposure was then repeated every 60 min up to 360 min. After each exposure, scanning was done with a flatbed scanner (EpsonES‐10000G, Seiko Epson Corp. Nagano, Japan) and images were acquired using Adobe Photoshop CS2 (Adobe Systems Inc., San Jose, CA). Scanning was done with 48 bit, 100 dpi resolution, and RGB mode with a PPC film (CR‐PP686) (3M Co., St. Paul, MN), using a liquid‐crystal protective film (LCD‐230W) (Sanwa Supply Inc., Okayama, Japan) to prevent moiré artifacts (Newton's rings).^(15)^ The Gafchromic films were always scanned in the same direction (portrait). The films were placed near the center of the scanner and reproducibly in the same position each time to avoid scanner nonuniformity errors, as shown in Fig. 1.^(16)^

Procedures for preparations and Gafchromic film scanning were done in a room in which the temperature change was within 21°C to 25°C.

### D. Image analysis

Only the image of the red channel was used for the analysis of the image. For a scanned image, the image data for the amount of change in pixel values without UV exposure was determined to identify the difference in pixel values changes when an image was UV irradiated.

A region of interest (ROI) one‐half inch in diameter was placed at the center of a Gafchromic film and the mean ± standard deviation (SD) pixel value was measured (Fig. 1). Graphs were prepared with UV exposure times vs. pixel values, from which the UV light that provided the most efficient density change was chosen. Digitized image data were analyzed with ImageJ, version 1.44 (National Institutes of Health, Bethesda, MD), image analysis software for Macintosh.

## III. RESULTS

The exposure strengths of UV‐A, UV‐B, and UV‐C sources were $0.074\;\text{mW}/{\text{cm}}^{2}$, $0.050\;\text{mW}/{\text{cm}}^{2}$, and $0.091\;\text{mW}/{\text{cm}}^{2}$, respectively. Total UV exposures with UV‐A, UV‐B, and UV‐C sources for 360 min were $1604.88\;\text{mJ}/{\text{cm}}^{2}$, $1086.88\;\text{mJ}/{\text{cm}}^{2}$, and $1965.60\;\text{mJ}/{\text{cm}}^{2}$, respectively (Table 1).

**Figure 3:**
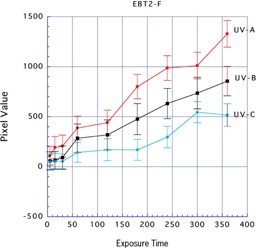
Results of three types of UV exposure to the front side of Gafchromic EBT2.

The UV‐A, UV‐B, and UV‐C were used to irradiate the front side of Gafchromic EBT2. When UV‐A was used for exposure for 360 min, the mean ±SD pixel values were highest, at 1,328.99±133.15. The mean pixel values with UV‐B and UV‐C were 856.11±146.59 and 516.77±112.76, respectively. These reactions were not accepted. That is, in the front side of Gafchromic EBT2, a reaction was not detected for 360 min with UV‐A light at $0.074\;\text{mW}/{\text{cm}}^{2}$ ($1,604.88\;\text{mJ}/{\text{cm}}^{2}$) because of the UV ray protective layer (Fig. 3).

**Table 1:** Three kinds of UV rays strength that irradiated Gafchromic films

*Exposure Time (min)*	*UV‐A*	*UV‐B*	*UV‐C*
5	22.29	15.09	27.30
10	44.58	30.18	54.60
15	66.87	45.27	81.90
30	133.74	90.54	163.80
60	267.48	181.08	327.60
120	534.96	362.16	655.20
180	802.44	543.24	982.80
240	1,069.92	724.32	1,310.40
300	1,337.40	905.40	1,638.00
360	1,604.88	1,086.48	1,965.60

UV Strength ($\text{mJ}/{\text{cm}}^{2}=\text{mW}/{\text{cm}}^{2}\times s$)

**Figure 4:**
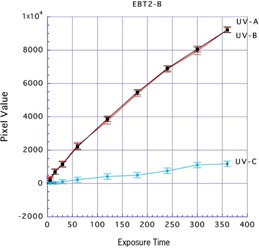
Results of three types of UV exposure to the back side of Gafchromic EBT2.

Next, the UV was used to irradiate the back side of Gafchromic EBT2. The mean ± SD pixel values after exposure with UV‐A, UV‐B, and UV‐C for 360 min were 9,226.63±182.04, 9,219.77±153.32, and 1,177.51±162.77, respectively. The reactions due to UV‐A and UV‐B were high, which provided effective results, whereas this was not the case with UV‐C (Fig. 4). Based on these results, UV exposure of Gafchromic EBT2 was effectively achieved by irradiating with UV‐A or UV‐B to the back side of this film that was not UV ray protected.

**Figure 5:**
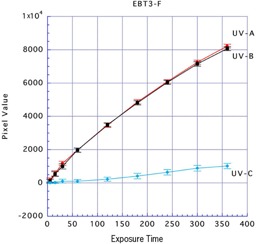
Results of three types of UV exposure to the front side of Gafchromic EBT3.

**Figure 6:**
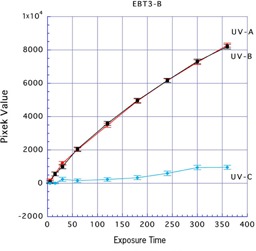
Results of three types of UV exposure to the back side of Gafchromic EBT3.

For Gafchromic EBT3, the mean ±SD pixel values with UV‐A, UV‐B, and UV‐C sources were 8,222.01±109.94, 8,071.71±119.92, and 1,012.39±159.20, respectively, after exposure for 360 min to the front side; and were 8,290.66±128.08, 8,198.06±138.35, and 957.17±126.90, respectively, after exposure for 360 min to the back side. Gafchromic EBT3 is designed with structural front and back symmetry. Thus, with exposure with UV‐A and UV‐B, there were approximately equal reactions on both sides of this film, whereas UV‐C resulted in very minimal reactions (Figs. 5 and 6).

Comparing the types of UV rays, there were reactions in the front and back sides of Gafchromic EBT3 and the back side of Gafchromic EBT2 with UV‐A. There were also reactions in the front and back sides of Gafchromic EBT3 and the back side of Gafchromic EBT2 with UV‐B. However, UV‐C resulted in minimal reactions on either side of Gafchromic EBT2 and EBT3. From these results, we considered that UV‐A or UV‐B could be used to effectively irradiate Gafchromic EBT2 and EBT3.

## IV. DISCUSSION

### A. Film Reactions

We found different results when UV exposure was applied to the front or to the back side of Gafchromic EBT2. Because UV ray protection is present on the front side of this film, reactions were low for 360 min exposure to any type of UV source. However, by applying exposure to the back side, very high reactions were generated. The mean pixel values were 1,328.995 in the front and 9,226.631 in the back side when this film was irradiated with UV‐A for 360 min, which was 6.94 times higher at the back side. The mean pixel value was 856.111 in the front side and 9,219.774 in the back side with UV‐B: 10.769 times higher at the back side.

When Gafchromic EBT3 was irradiated for 360 min with UV‐A and UV‐B, high reactions were generated that were equal at both the front and the back sides, with mean pixel values of 8,222.005 (front) and 8,290.660 (back) with UV‐A and 8,071.713 (front) and 8,198.057 (back) with UV‐B. When both sides of Gafchromic EBT3 were compared with the back side of Gafchromic EBT2, the back side of Gafchromic EBT2 showed a higher reaction. For an index of the exposure strength, the exposure time was set and an exposure dose was measured.

### B. UV protection layer

The front of Gafchromic EBT2 is UV‐ray protected; therefore, there were minimal reactions. However, UV‐A resulted in greater reactions than those with UV‐B and UV‐C. The pixel values were 1,328.995±133.151, 856.111±146.588, and 516.766±112.762 after exposure for 360 min with UV‐A, UV‐B, and UV‐C, respectively. That is, a Gafchromic film with a UV ray protection layer can be used by increasing the exposure strength.

### C. UV protection for humans

The wavelengths of UV‐A are from 315 to 400 nm, those of UV‐B are from 280 to 315 nm, and those of UV‐C are from 200 to 280 nm. UV rays may affect the human body. A UV exposure box was made with an acrylic board to cut UV rays to prevent this. There was no leakage of UV rays to the outside of this box.

### D. Yellow dye in Gafchromic EBT2

One purpose for using a yellow dye in Gafchromic EBT2 and EBT3 films is that variations in active layer thickness results in density changes and the dye is used for nonuniformity corrections. Radiation dose information is provided by a red channel, and information on the degree of uniformity is provided by a blue channel, which information is used for nonuniformity corrections.^16^, ^17^ However, the uniformity of Gafchromic EBT2 in a single red channel using a double exposure technique (pre‐exposure technique) was equal to that of a triple‐channel method.^(8)^ Therefore, thickness‐unevenness of the active layer was enhanced by uniformly irradiating with UV rays, such as UV exposure to Gafchromic EBT film.^(7)^

### E. UV strength

Because we irradiated at a distance of 72 cm using a 10 W fluorescent tube, relatively long‐time exposure was necessary for an increase in density. When there was some increase in density even with exposure for 5 min and on comparison to dose data for nonexposure a difference was observed, a reaction due to UV rays could be expressed. However, the most suitable exposure dose for EBT2 and EBT3 films remains to be determined. For UV lamps with high exposure strengths, exposure can be done within a short time. Thus, it is necessary to determine the most suitable exposure dose or exposure time.

In this study, the sensitivity of Gafchromic film for UV rays was measured; the most suitable UV ray strength should be evaluated in future studies.

### F. Scanning

Peculiar irregularities with quite a few scanners may occur during image scanning. In addition, these irregularities may occur because a protective film of liquid crystals and PPC are spread for moiré (Newton's rings) reduction between the glass surface of a scanner and a Gafchromic film. However, these irregularities can be reduced by making a subtraction, and only the changes that occurred will be expressed by UV exposure.

### G. UV wavelengths

Because different UV‐ray fluorescent tube types were used in this study, the wavelengths emitted had certain peaks and widths. Our results were best in terms of sensitivity with a UV lamp with a UV‐A wavelength of 365 nm. However, UV‐A wavelengths vary in the range of 315−400 nm and it could not be judged whether the UV rays with the UV lamp we used were the most efficient. It will be necessary to investigate this using a lamp that emits UV rays with wavelengths over a smaller range. It will also be necessary to conduct experiments using a UV ray apparatus (e.g., LED) that generates a specific wavelength to identify the required wavelength.

### H. Optical density increases

At certain times after exposure began there was an increase in optical density.^(16)^ Because this study was a evaluation of nonuniformity, density increase after the exposure was not considered.

## V. CONCLUSIONS

The UV rays that Gafchromic EBT3 reacted to most were UV‐A and UV‐B. Both sides of this film provided equal results. Both the front and back sides reacted to UV‐A and UV‐B, whereas for Gafchromic EBT2, the reactions were different. Even if exposure was to the front side of Gafchromic EBT2 with UV‐ray protection, it exhibited a reaction when using a 10W UV tube. Based on these results, using UV exposure, nonuniformities are emphasized in addition to the yellow dye. Uniform exposure with UV rays provided for using a Gafchromic film with a large area of UV rays could be used as a substitute for a double‐exposure technique with X‐rays. Thus, the precision of the measurement of X‐rays dose by a Gafchromic film would be improved.

## ACKNOWLEDGMENTS

This study was supported by JSPS KAKENHI Grant Numbers 26460740.

## References

[1] Gotanda R , Katsuda T , Gotanda T , et al. Computed tomography phantom for radiochromic film dosimetry. Australas Phys Eng Sci Med. 2007;30(3):194–99. 18044303 10.1007/BF03178426

[2] Katsuda T and Gotanda R . University Okayama University Corp. Dose measuring method and phantom, and x‐ray image picking‐up device used for the dose measuring method. WIPO patent application WO/2008/087952. 2008.

[3] Zhu Y , Kirov AS , Mishra V , Meigooni AS , Williamson JF . Quantitative evaluation of radiochromic film response for two‐dimensional dosimetry. Med Phys. 1997;24(2):223–31. 9048362 10.1118/1.598068

[4] Butson ET , Cheung T , Yu PK , Butson MJ . Measuring solar UV radiation with EBT radiochromic film. Phys Med Biol. 2010;55(20):N487–93. 20858922 10.1088/0031-9155/55/20/N01

[5] Butson ET , Yu PK , Butson MJ . Solar ultraviolet radiation response of EBT2 Gafchromic, radiochromic film. Phys Med Biol. 2013;58(21):N287‐94. 24113466 10.1088/0031-9155/58/21/N287

[6] Gafchromic EBT, self‐developing film for radiotherapy dosimetry. Wayne, NJ: International Specialty Products; 2005.

[7] Katsuda T , Gotanda R , Gotanda T , et al. Reducing non‐uniformity error of radiochromic film in the diagnostic range by ultraviolet exposure: preliminary study. IFMBE Proceedings. 2009;25(3):227–30.

[8] van Hoof SJ , Granton PV , Landry G , Podesta M , Verhaegen F . Evaluation of a novel triple‐channel radiochromic film analysis procedure using EBT2. Phys Med Biol. 2012;57(13):4353–68. 22705890 10.1088/0031-9155/57/13/4353

[9] ISO 21348: 2007. Space environment (natural and artificial) – process for determining solar irradiances. Geneva: International Organization for Standardization; 2007.

[10] Gotanda T , Katsuda T , Akagawa T , et al. Evaluation of Gafchromic EBT2 dosimetry for the low dose range using a flat‐bed scanner with the reflection mode. Australas Phys Eng Sci Med. 2013;36(1):59–63. 23479183 10.1007/s13246-013-0187-z

[11] Ebraheem S , Abdel‐Fattah AA , Said FI , Ali ZI . Polymer‐based triphenyl tetrazolium chloride films for ultraviolet radiation monitoring. Radiat Phys Chem. 2000;57(2):195–202.

[12] Abdel‐Fattah AA , Hegazy EA , el‐Din HE . Thymol‐blue dyed poly (vinyl butyral) film for monitoring ultraviolet radiation. J Photoch Photobio A. 2000;137(1):37–43.

[13] de Gruijl FR . Skin Cancer and Solar UV Radiation. Eur J Cancer. 1999;35(14):2003–09. 10711242 10.1016/s0959-8049(99)00283-x

[14] de Gruijl FR , van Kranen HJ , Mullenders LHF . UV‐induced DNA damage, repair, mutations and oncogenic pathways in skin cancer. J Photoch Photobio B. 2001;63(1–3):19–27. 10.1016/s1011-1344(01)00199-3 11684448

[15] Gotanda T , Katsuda T , Gotanda R , et al. Evaluation of effective energy using radiochromic film and a step‐shaped aluminum filter. Australas Phys Eng Sci Med. 2011;34(2): 213–22. 21437731 10.1007/s13246-011-0068-2

[16] Lewis DF . Practical Guide to Radiochromic Film EBT2/EBT3. 2012 http://www.filmqapro.com/Documents/Lewis_Radiochromic_Film_20120209.pdf. Accessed April 15, 2015.

[17] Micke A , Lewis DF , Yu X . Multichannel film dosimetry with nonuniformity correction. Med Phys. 2011;38(5):2523–34. 21776787 10.1118/1.3576105

